# Physical fitness and training factors associated with injury risk in aerobic gymnastics: a systematic review

**DOI:** 10.3389/fpubh.2026.1803284

**Published:** 2026-03-03

**Authors:** Wenxin Yang

**Affiliations:** Chengdu Sport University, Chengdu, China

**Keywords:** aerobic gymnasts, gymnastics, injury risk, risk factors, training load

## Abstract

**Objectives:**

Aerobic gymnastics involves repeated high-impact technical elements, yet discipline-specific evidence linking modifiable physical fitness and training exposures to injury risk remains fragmented. Thus, the aim of this systematic review was to synthesize evidence on physical fitness attributes and training-related factors associated with injury risk in aerobic gymnasts, and to appraise study designs, injury definitions, and measurement approaches.

**Methods:**

A systematic search was conducted in PubMed, Scopus, and Web of Science Core Collection. It were eligible studies including aerobic gymnasts that reported quantitative associations between modifiable fitness and/or training exposures and injury outcomes. Risk of bias was executed with QUIPS.

**Results:**

Twelve studies were deemed eligible. Injury/pain burden was substantial and typically involved the lower limbs and, in some samples, the lumbar region. Greater exposure (training volume/competition density) was consistently associated with injury occurrence in some studies, and lower-limb alignment/loading-distribution measures differed between injured and uninjured aerobic gymnasts, although screening models showed limited sensitivity. Psychosocial factors and limited prevention/healthcare uptake were also reported.

**Conclusion:**

Current evidence suggests injury risk in aerobic gymnastics is multifactorial, with exposure and selected alignment/loading measures as recurrent correlates. Prospective, standardized surveillance and robust analytic approaches are needed.

**Systematic review registration:**

https://osf.io/d6pmj.

## 1 Introduction

Aerobic gymnastics (also termed competitive aerobics) is a gymnastics discipline in which athletes perform short, choreographed routines to music that combine continuous, high-intensity locomotor sequences with sport-specific technical elements (1). The routines feature repeated explosive jumps, rapid changes of direction and rotation, and intermittent strength elements, exposing athletes to frequent take-off and landing cycles that concentrate mechanical loading in the lower limbs (2, 3). From an injury-mechanism perspective, the most salient training exposures likely include repeated plyometric landings and rebound jumps, high-cadence rhythmic locomotor sequences that accumulate fatigue, and technical elements with asymmetrical take-off/landing demands that can bias limb loading (4). Across jumping/landing sports, external load (ground-reaction forces, contact counts) and internal load (perceived exertion, physiological strain) jointly shape tissue stress and recovery requirements, providing a practical basis for monitoring both how much and how hard training is performed (5). Physiological profiling using routine-like protocols indicates a substantial anaerobic contribution alongside high cardiometabolic stress, underscoring the importance of power, strength-endurance, and fatigue resistance for performance (1). Longitudinal evidence in youth aerobic gymnasts suggests that regular discipline-specific training improves performance-related capacities (e.g., dynamic balance and lower-limb strength endurance), while adaptations may be less pronounced for some neuromuscular qualities such as explosive power without targeted programming (6).

Across gymnastics disciplines, athletes demonstrate a considerable injury burden, with surveillance systems and systematic reviews describing frequent injuries and a prominent contribution of overuse conditions (7–9). In female gymnastics, the majority of athletes may sustain at least one injury per season, and the ankle, knee, and lumbar region are repeatedly reported among the most affected sites (10, 11). In this context injury and pain/musculoskeletal problems are related but not interchangeable constructs, since pain/problems may represent early or non–time-loss manifestations along a health-problem continuum, whereas injury is typically considered via time-loss, medical-attention, or defined reportable events. Impact and landing tasks are consistently highlighted as potentially injurious exposures in gymnastics, given the high ground-reaction forces and joint loading demands associated with repeated dismounts and jump-land actions (12, 13). Although multisport surveillance has captured injuries in aerobic gymnastics, event-level reporting provides limited discipline-specific detail to isolate modifiable risk factors for aerobic gymnasts (14).

Within gymnastics, researchers have evaluated whether physical fitness attributes (e.g., flexibility, strength, balance, and movement quality) assessed through screening tools can predict future injury, yet overall findings remain heterogeneous and tool performance varies substantially across studies (15). In this context, physical fitness attributes denote modifiable capacities (e.g., neuromuscular performance, mobility, motor control, and body composition), whereas physical characteristics includes largely non-modifiable descriptors that may act as covariates or effect modifiers rather than intervention targets. Prospective evidence from rhythmic gymnastics indicates that flexibility characteristics can be associated with injury occurrence, supporting the concept that both insufficient and excessive range of motion may influence tissue loading and injury susceptibility depending on context (16). Biomechanics research in gymnastic-style landings provides plausible mechanistic links between modifiable neuromuscular qualities (e.g., strength, stiffness regulation, and stability control) and lower-limb loading patterns that may elevate injury risk (13, 17). In competitive aerobics athletes, ability-level differences in rotational jump landings include distinct lower-limb kinematics and kinetics, suggesting that sport-specific fitness and technique may meaningfully alter exposure to potentially injurious loads (2). In parallel, training exposures can be conceptualized not only as quantity (volume, frequency, density) but also as qualitative load features, such as routine technical difficulty, asymmetry of choreography, and repetition of high-impact elements, that can redistribute tissue stress and may be especially relevant in aerobic gymnastics.

Gymnastics participation commonly begins early and involves high weekly training volumes with repeated high-impact skill practice, which can amplify cumulative tissue loading during periods of rapid growth and maturation (18, 19). In youth gymnastic pathways, weekly training commonly reaches double-digit hours and may exceed 15 h/week in some cohorts, particularly during early adolescence (19). Across training contexts, greater total exercise volume and rapid load increases are consistently associated with higher injury risk, while appropriately progressed chronic loads may support protective fitness adaptations (20, 21). Conceptually, injury risk in aerobic gymnastics can be framed as an interaction between exposure (dose: training volume, competition density, technical repetition) capacity (strength, power, neuromuscular control, mobility), and recovery/context (psychological stress, sleep, access to care, surfaces/equipment) (22). Workload–injury etiology models further propose that workloads contribute to injury risk through exposure, fatigue, and fitness pathways, reinforcing the importance of monitoring both absolute load and change in load over time (4, 23). In gymnastics, internal load parameters have been associated with injury incidence, and in young aerobic gymnasts congested training periods have been linked to strength decrements and increased landing forces that could plausibly heighten injury risk (3, 24). Accordingly, aerobic gymnastics should be framed as a discipline in which both cumulative volume and short-term load fluctuations are plausible drivers of injury susceptibility (4).

While injury epidemiology and risk-factor evidence are well developed in artistic gymnastics and are expanding in other disciplines, syntheses rarely isolate aerobic gymnastics, limiting the transferability of prevention insights to its distinct performance demands (9, 15). Aerobic gymnastics uniquely combines sustained, high-cadence rhythmic movement with repeated plyometric landings and rapid directional changes on a sprung floor, yielding a mixed metabolic–mechanical loading profile that is not well represented by artistic- or rhythmic-gymnastics evidence alone (5). Because injury etiology is dynamic and sport-context dependent, discipline-specific syntheses are required to avoid misapplication of screening and load-management heuristics derived from dissimilar exposure patterns (22). Therefore, consolidating aerobic-gymnastics–specific evidence is necessary to define which modifiable exposures and capacities most consistently co-vary with injury burden in this sport.

The aerobic gymnastics literature has more commonly emphasized performance profiling and biomechanical characteristics than injury outcomes, and the evidence linking modifiable physical fitness and training exposures to injury risk remains comparatively fragmented (1–3). A systematic synthesis is therefore warranted to consolidate the available evidence, identify consistent fitness- and training-related correlates of injury risk in aerobic gymnasts, and define priorities for methodologically robust prospective research in this discipline. Therefore, the objectives of this systematic review are: (i) to synthesize the evidence on physical fitness attributes associated with injury risk in aerobic gymnasts, and (ii) to synthesize the evidence on training-related factors (e.g., exposure, load, and training-period characteristics) associated with injury risk in aerobic gymnasts. A secondary objective is to critically appraise study designs, injury definitions, and measurement approaches used in this field to inform future research and injury-prevention practice in aerobic gymnastics.

## 2 Methods

The systematic review methods and reporting were developed and written in accordance with the PRISMA 2020 statement and the PRISMA 2020 expanded checklist (25). A review protocol was prepared *a priori* to define the objectives, eligibility criteria, search strategy, and planned methods for study selection, data extraction, risk of bias assessment, and synthesis. The protocol was published at the Open Science Framework webpage with the code number osf.io/d6pmj at 25/01/2026.

### 2.1 Eligibility criteria

Studies were eligible if they included aerobic gymnasts (any sex, age group, or competitive level) and evaluated associations between modifiable physical fitness characteristics and/or training-related factors and injury outcomes. Eligible physical fitness exposures included, but were not limited to, neuromuscular performance and movement qualities such as strength, power, strength-endurance, balance, flexibility or range of motion, motor control, and body composition or anthropometrics when analyzed as potentially modifiable correlates. Eligible training-related exposures included measures of training exposure, frequency, volume, intensity, internal and/or external training load, training monotony/strain, periodization characteristics, and competition density or congested schedules when examined in relation to injury outcomes.

The primary outcome domain was injury occurrence in aerobic gymnasts, operationalized as any reported injury incidence, prevalence, rate, risk, or time-to-event, including acute and overuse conditions, regardless of whether the study used a time-loss, medical-attention, or any-complaint definition. For clarity, we treated risk as the probability of a new injury over a defined follow-up (prospective designs), and correlates as cross-sectional or retrospective associations with injury history or current problems (22). We also conceptualized pain and musculoskeletal problems as potentially overlapping with injury outcomes, consistent with health-problem surveillance frameworks that capture time-loss and non–time-loss conditions. Studies were required to report an injury outcome quantitatively and to present an association between at least one eligible exposure and injury, or sufficient data to derive an association. Observational designs (prospective or retrospective cohort, case–control, and cross-sectional studies) were eligible. Controlled trials were eligible only if they reported injury outcomes and provided analyzable associations between baseline or training-related exposures and subsequent injury. Case reports, case series with very small samples, narrative reviews, systematic reviews, conference abstracts without sufficient methodological detail, editorials, and opinion pieces were excluded.

No restrictions were applied to publication year. Reports were considered regardless of language. Where a full text required translation, translation was undertaken using automated translation with verification by a professional fluent in the language where feasible.

### 2.2 Information sources

PubMed, Scopus, and Web of Science Core Collection were searched on 26 January 2026, and this date was recorded as the last search date for each source. In addition, the reference lists of all included studies and of relevant review articles identified during screening were examined to identify additional eligible studies.

### 2.3 Search strategy

Search strategies were developed to capture three two core concepts: aerobic gymnastics (including common synonyms such as sport aerobics and competitive aerobics), and injury outcomes. The PubMed strategy used a combination of keywords and, where applicable, controlled vocabulary terms; analogous term sets were adapted for Scopus and Web of Science Core Collection to reflect platform-specific syntax and field tags. No date limits were applied, and language limits were not used. The specific search strategy is shown in Table 1.

**Table 1:** Search strategy.

Domains	Search specificities	Search terms
Aerobic gymnastics	Title, abstract and keywords (topic)	“aerobic gymnastics” OR “aerobic gymnasts” OR “sport aerobics” OR “competitive aerobics” OR “gymnastics” OR “gymnasts”
		AND
Injury	Title, abstract and keywords (topic)	Injury OR injuries OR strain OR time-loss

### 2.4 Selection process

All records retrieved from the searches were exported to Zotero for de-duplication, after which the deduplicated library was uploaded to a systematic review screening platform for study selection. The author screened titles and abstracts against the eligibility criteria, with an independent, blinded external expert screening in parallel. Both screened all records at title/abstract stage and all retrieved full texts in duplicate, independently and blinded to each other’s decisions. Inter-rater agreement was quantified as percent agreement, showing 93%. Full texts were retrieved for all records deemed potentially eligible or unclear, and the author and the blinded external expert independently assessed full texts for inclusion. Decisions were cross-checked, and discrepancies at either stage were resolved through discussion and consensus; when agreement could not be reached, a second independent blinded external expert adjudicated. When information required to determine eligibility was missing or ambiguously reported, attempts were made to clarify eligibility by contacting study authors.

### 2.5 Data items

Data were sought for injury outcomes at the broadest level reported by each study, including overall injury occurrence and, where available, stratified outcomes such as acute versus overuse injury, anatomical region-specific injury, time-loss injury, and injury severity metrics. When studies reported multiple compatible operationalisations of injury (for example, both medical-attention and time-loss outcomes), all eligible outcomes were extracted and clearly mapped to their definitions and observation windows. If multiple time points were reported, the earliest time point reflecting prospective injury occurrence during follow-up was prioritized for primary extraction, while additional time points were retained for contextual interpretation.

Data were extracted on participant characteristics (sample size, sex distribution, age, competitive level, training history, and growth/maturation indicators when available), study and season context, and methodological features relevant to internal and external validity. Data were extracted on exposure measurement methods (test protocols for fitness variables, monitoring methods for training variables, timing of exposure assessment relative to injury surveillance, and units of measurement). Data were also collected on injury surveillance procedures (injury definition, method of ascertainment, reporter type, and exposure-time denominator when used). Data extraction was performed by the author and independently verified by a blinded external expert using a extraction form. Discrepancies were resolved by consensus with reference to the full text.

### 2.6 Study risk of bias assessment

We evaluated internal validity for each included study with the Quality in Prognosis Studies (QUIPS) instrument (26), which is designed for reviews examining prognostic or risk-factor relationships between an exposure and subsequent outcomes. The author and an independent, blinded external expert appraised all studies in parallel, rating the likelihood of bias within each QUIPS domain: participant selection, loss to follow-up, measurement of the prognostic factor, measurement of outcomes, handling of confounding, and the adequacy of statistical methods and reporting. Domain ratings were then combined into an overall risk-of-bias classification using predefined decision criteria that placed particular weight on identification and adjustment for key confounders and the validity and reliability of measurements for discipline-relevant exposures (e.g., training volume, competition density, internal load indices, and biomechanical/alignment measures) and injury outcomes. Because several included studies were cross-sectional or retrospective survey designs, we applied QUIPS using design-adapted decision rules: (i) for study attrition, one-time surveys were rated based on participation/non-response bias and completeness of key fields (rather than loss to follow-up), and (ii) for prognostic factor measurement, we rated the reliability/validity of retrospective exposure measurement and the risk of differential recall (particularly when exposures and outcomes were self-reported over long windows). Assessments were cross-checked, and any discrepancies were resolved through discussion to reach consensus, with adjudication by a second independent blinded expert when necessary.

### 2.7 Effect measures and synthesis methods

For each exposure–injury association, effect estimates were extracted as reported, including odds ratios, risk ratios, hazard ratios, incidence rate ratios, regression coefficients, correlation coefficients, or other appropriate measures, along with corresponding precision estimates such as confidence intervals, standard errors, or *p*-values. Where necessary for interpretability across studies, effect estimates were harmonized in direction so that the referent category and the direction of higher risk were explicitly stated. *A priori* selection rules were applied when multiple eligible estimates were available. We prioritized adjusted models over unadjusted models when the covariate set included at least age/sex and prior injury or exposure; then we prioritized time-loss outcomes over any-complaint outcomes when both were available because of greater clinical specificity, while retaining any-complaint outcomes for burden interpretation; and finally when multiple operationalisations of the same construct were reported, we prioritized the measure with the clearest definition and greatest sport-specific relevance.

Studies were first organized into prespecified synthesis groupings based on the primary exposure domain, separating physical fitness factors from training-related factors, and then further grouped by specific construct (for example, strength/power, flexibility, balance/motor control, or training load/exposure). Within each grouping, studies were also classified by design (prospective versus retrospective/cross-sectional) and by injury outcome definition (for example, any injury versus time-loss injury, and acute versus overuse where available) to support meaningful interpretation.

A structured narrative synthesis was undertaken, supported by detailed evidence tables that presented study characteristics, exposure operationalisation, injury definitions, analytical models, and extracted effect estimates with precision. When multiple studies evaluated conceptually similar exposures and comparable injury outcomes, consistency of direction and magnitude of associations was examined qualitatively, with explicit attention to study design and risk of bias when interpreting patterns. Quantitative pooling was considered inappropriate when fewer than three studies assessed a comparable exposure–outcome pair, when injury definitions were not compatible, when exposure units/operationalisations could not be harmonized, or when risk of bias was predominantly high in outcome measurement/confounding domains.

To prepare data for synthesis, units and operational definitions of exposures were recorded verbatim, and where studies reported multiple operationalisations of the same construct, the most sport-relevant or most clearly defined measure was prioritized for the primary narrative while retaining alternative measures for sensitivity of interpretation.

## 3 Results

### 3.1 Study selection

**Figure 1:**
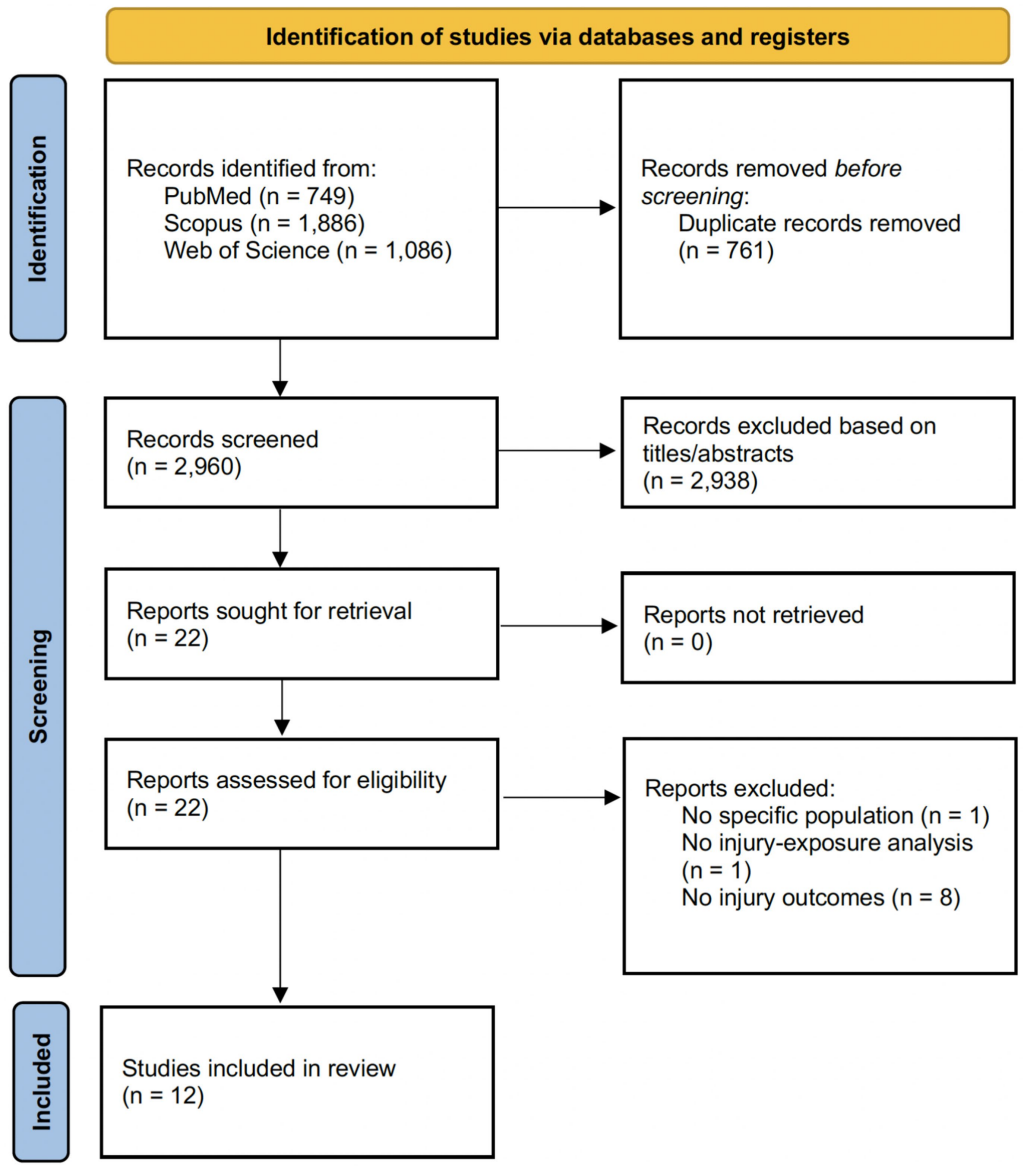
PRISMA flowchart.

Figure 1 presents the PRISMA 2020 flow diagram summarizing the study selection process. Database searching identified 3,721 records (PubMed, *n* = 749; Scopus, *n* = 1,886; Web of Science, *n* = 1,086). After removal of 761 duplicates, 2,960 records were screened by title and abstract, and 2,938 were excluded at this stage. Full texts were sought for 22 reports. Ten reports were excluded following full-text assessment due to no injury outcomes (*n* = 8), no specific population (*n* = 1), or no injury–exposure analysis (*n* = 1). In total, 12 studies met the inclusion criteria and were included in the systematic review.

### 3.2 Study characteristics

Table 2 summarizes the methodological characteristics of the included studies, presenting the design features.

**Table 2:** Methodological characteristics of included studies.

Study	Design	*N*	Sex	Age	Competitive level	Exposure domain	Exposure variables	Timing of exposure assessment	Injury definition	Outcome measure(s)	Ascertainment method & reporter	Outcome stratification collected
Abalo-Núñez et al. (29)	Retrospective cohort	40	Not reported	9–17 years; mean 12 ± 2.52	Competitive youth/junior; includes Spanish-team gymnasts (*n* = 12)	Training-related	Training load (days/week, hours/day, competitions); protective equipment; training surface; technical movements/work phase	End of season (retrospective over 2009–2010)	Damage/accident/incident during competition or training causing time away from competition, or ≥2 days training lost, or reduced load in ≥2 consecutive sessions	Injury occurrence and count during season; injury type and anatomical site; timing (e.g., work phase/month); associations with training factors	Self-reported via administered morbidity questionnaire (researcher-administered)	Category (Alevin/Youth/Junior); injury type (ligament/tendon/muscular/articular); body part; work phase/technical gesture; month; recurrence; protective equipment use; surface
Abalo et al. (34)	Quasi-experimental, retrospective longitudinal	73	Gymnasts: 45F/6M	6–23 years; Gymnasts 13.61 ± 4.59; Controls 14.59 ± 3.93	Competitive aerobic gymnastics (regional/national/international; not fully detailed in this paper’s methods)	Physical fitness (anthropometric/morpho-constitutional)	Q-angle (left/right; mean of records 1&2), bilateral weight-bearing (right/left; mean of records 1&2), thigh perimeter (right/left; mean of records 1&2), plus between-limb differences	Anthropometrics measured after consent (baseline) before season injury data were compiled; injury data collected retrospectively for 2009–2010 season	Injuries recorded via validated questionnaire-interview for the 2009–2010 season	Injuries during 2009–2010 season (injury/no injury; incidence described); injury type/location; logistic regression equation performance (specificity/sensitivity)	Retrospective questionnaire-interview on injuries/training (reporter not explicitly specified; athlete self-report via interview-questionnaire)	Group (gymnasts vs. controls); injury type; injury location/body region
Abalo-Núñez et al. (36)	Quasi-experimental, retrospective and longitudinal	73	Gymnasts: 45F/6M	6–23 years; Gymnasts 13.61 ± 4.59; Controls 14.59 ± 3.93	Gymnasts: 39.22% international, 56.86% national, 3.92% regional; Controls: 4.55% international, 45.45% national, 50% regional	Physical fitness (anthropometric)	Q angles (RQA/LQA, DQA); bilateral weight-bearing (BWBRL/BWBLL, DBWB); thigh perimeters (PRT/PLT, DPT); plus interaction with weight	Baseline anthropometrics (2 measurement days) prior to end-of-season injury ascertainment; injuries collected at end of season	Injury/no injury at end of season captured by validated questionnaire	Injury at end of season (binary); logistic regression prediction models (classification, ORs); also previous injury history and reinjury during season	End-of-season injury questionnaire (validated); athlete self-report via questionnaire administered by study team	Group (gymnasts vs. controls); previous injury status; training days; competition level; reinjury vs. new injury
Gonçalves et al. (27)	Cross-sectional	189	Mixed; 73.5% female (139), 26.5% male (50)	Adults; mean 21.2 ± 4.1 years	Elite/senior (world championship participants)	Both (intrinsic & extrinsic/training-related)	Sample data (age, sex, height, weight); AG experience; specialization age; training frequency/duration; competitions; categories; strength conditioning; prevention exercises; warm-up/cool-down; protective equipment; facilities/equipment; training load adaptation; balanced diet; psychological stress; recurrent injuries; other sport practice	Administered during WCH 2022 (after podium training) with retrospective recall of prior 12 months/2021–2022 season	Gymnasts reported injuries in the last 12 months and characterized number/type/tissue, body region, and mechanism	12-month injury prevalence and mean number of injuries; injury type/tissue and body region distributions; mechanisms; Pearson correlations between risk factors and injury presence/absence	Athlete self-report via electronic questionnaire (Survio) completed on personal devices; study team supervision during completion	Acute vs. overuse vs. both; place (training/competition); contact (alone/another athlete/object); tissue/injury type categories; body part/region; number of body parts; recurrence; risk-factor item responses (stress, protective equipment, facilities, etc.)
Hassmannová et al. (30)	Cross-sectional	58	Female only	12–15 years; mean 13.52 ± 1.24	Elite youth (high competition level)	Training-related	Training intensity; sport achievements (medal position vs. non-medal); other motion activities; approach to musculoskeletal problems (immediate professional help); physiotherapy prevention (primary and secondary/follow-up)	Interview conducted toward/after end of younger school age (participants aged 12–15), retrospectively recording musculoskeletal problems and care during younger school-age period	Injury: musculoskeletal condition preventing sports activity; only training-related pain/injuries recorded	Occurrence/prevalence of training-related musculoskeletal problems (pain and injuries); affected body areas; relationship with performance level; uptake of medical/physiotherapy care and prevention	Child self-report (with parent present) during structured interview; information recorded by researcher in questionnaire	Pain vs. injury; body area (lower limbs/back/upper limbs; specific joints); training intensity category; performance level (medal vs. non-medal); other activities; professional attention vs. primary vs. secondary physiotherapy prevention
Hassmannová et al. (31)	Cross-sectional	58	Female only	12–15 years; mean 13.52 ± 1.24	Elite youth (high competition level)	Training-related	Training intensity; sport achievements; other motion activities; training-related pain/injury; approach to problems (doctor/physio immediately after onset); physiotherapeutic prevention (primary and secondary/follow-up)	Interviews conducted Dec 2017–Jan 2018 toward/after end of younger school age; retrospective recording of younger school-age period	Injury: musculoskeletal condition preventing sports activity; only training-related pain/injuries recorded	Occurrence/prevalence of musculoskeletal problems (pain and injuries) and most commonly affected areas (e.g., ankle/knee); association with performance level; professional monitoring and physiotherapy prevention uptake	Child self-report (with parent present) during structured interview; information recorded by researcher in questionnaire	Pain vs. injury; body region and specific joints; training intensity category; performance (medal vs. non-medal); other motion activities; professional attention vs. primary vs. secondary physiotherapy prevention
Li et al. (38)	Computational thermal modeling/machine-learning early-warning model with experimental infrared thermal imaging data	3	Female (*n* = 3 in experiment)	18 years (*n* = 3)	Competitive aerobics athletes (high-level/elite)	Physiological monitoring/biomechanical-thermal indicators	Infrared thermal image temperature distribution. “abnormal hot areas” indicating fatigue/overuse; mapped injury data converted into early-warning classification levels	During training and competition (real-time monitoring implied)	“actual damage cases” and “sports injury data” used as labels for early-warning model	Model performance (accuracy, MSE, Pearson correlation); sensitivity/specificity for predicting injury risk; identification of abnormal thermal regions/early-warning levels	Thermal imaging + algorithmic classification; source/validation of “actual damage cases” not described	Gender-level injury incidence reported; limited additional stratification reported (details of injury labels not provided)
Purnell et al. (33)	Retrospective injury and training survey	73	Female: 69; Male: 4	Range 8–26 years; females mean 13.4 (SD 3.6) years; males mean 20.5 (SD 4.2) years	Recreational and competitive; competitive levels reported	Training load/participation; individual characteristics; role/skill-type exposure	Training volume (hours/week current and at ages 11–15); years of acrobatics training; training components (conditioning, tumbling, group skills); competitive level; role/position (base/top/middle) and skill type (static/balance vs. dynamic); anthropometrics (height/weight/BMI); menstrual history; other sports training history	Single survey administration; retrospective reporting of training (including past years/h) and injuries	Chronic injury: currently affects training/performance with continuing problems ≥3 months	Injury occurrence (past 12 months; past 6 months reported in age-group table); chronic injury prevalence; total injury history; injury incidence rate per 1,000 h (computed from reported training hours); injury site/diagnosis/mechanism; treatment and time to return to training; perceived causes	Self-report questionnaire (athletes; parents/guardians assisted primary-school-aged participants)	Age groups (≤12 vs. ≥ 13); competitive level; role/skill type (group skills; base/top/middle; static vs. dynamic); injury onset type (acute vs. gradual/chronic); anatomical site and diagnosis; treatment and recovery duration
Abalo et al. (28)	Descriptive study with cross-sectional administration of a retrospective interview-questionnaire	42	Female: 33 (79%); Male: 9 (21%)	Mean 18.78 years (SD 5.84); junior and senior categories	Elite national (many international); junior and senior competitors at Spanish Championship	Training characteristics/environment (volume, planning, protection, surface)	Time of practice; hours/day and days/week; training planning (plan/no plan); safety/protective equipment use; regulatory vs. non-regulatory floor/surface (and related equipment/material)	Single administration during pre-competition training sessions; retrospective reporting of prior season injuries/training	Not explicitly stated; athletes reported injuries experienced during the 2010–2011 season, which were then classified by tissue type and functional severity category	Injury presence/number; injury type and location; severity (functional categories); cause and sequelae; whether occurred during training vs. competition; medical consultation, rehabilitation needs, and sequelae	Self-report via interview-questionnaire administered by the same researcher; informed consent obtained	Category (junior vs. senior) and sex; injury type, location, severity; training vs. competition; use of protection and surface-related variables; medical care, rehabilitation, and sequelae
Sastre-Munar et al. (32)	Observational cross-sectional	160	Predominantly female: 150/160 (93.8%); male: 10/160 (6.2%)	Mean 16.9 ± 3.0 years (analyzed sample)	Elite vs. non-elite (elite = competed in international and national finals; 16.9% elite overall)	Psychological & training-related (with pain status)	Pain catastrophizing (PCS total + subscales: rumination, helplessness, magnification); pain intensity (NRS); training volume (h/week); discipline; competition level; age; sport experience	Single administration at end of season (May–July 2021); injury questions refer to current season; pain/NRS and PCS captured at survey	Injury related to sport/exercise with consequent disruption in sport/exercise for ≥7 days	Injury prevalence in current season (injured yes/no) and number of injuries; pain presence and NRS; PCS total and subscales; associations between PCS, pain, injuries, and training characteristics	Athlete self-report (online questionnaire; informed consent by athletes/guardians)	Stratified by discipline (artistic vs. rhythmic), sex, elite vs. non-elite; injured vs. non-injured; pain vs. no pain; PCS subscales
Xiong et al. (40)	Algorithm development/validation study	200	Mixed (male and female groups by aerobics level)	College age; group mean ages reported 18.9–22.1 years	Competitive aerobics levels (Master/Level 1/Level 2) plus ordinary students; groups stratified by gender and aerobics level	Movement screening & spatiotemporal movement data	FMS score (functional movement screening); aerobics action spatiotemporal features from multiple detection points; big-data features used for deep-learning prediction (details limited)	Baseline testing and model training/testing sessions (timing and number of sessions not reported)	Not explicitly defined; outcome framed as “sports injury risk” probability	Predicted sports-injury risk probability (prediction curve vs. ‘true’ curve); model performance/accuracy comparisons including ablation without FMS	Algorithmic outputs compared with ‘true’/reference curve derived from data included within the article (source of injury labels not described)	Model comparison with vs. without FMS (ablation); results presented over sequential periods
Zhu et al. (39)	Algorithm development/experimental study	58	Female only (58 girls analyzed; 2 excluded due to acute injury at start from an initial 60)	Not reported (freshman/sophomore college students)	Competitive level not clearly specified	Movement-performance imagery/computer-vision classification	Aerobics injury categories for classification (joint strain, joint sprain, muscle strain); risk grouping (high-risk vs. low-risk) and corrective training intervention mentioned (group definitions not fully described)	During the experimental training/testing phase (1-week); baseline screening excluded participants with acute injury	Acute sports injuries; model focuses on closed-injury types (joint strain, joint sprain, muscle strain); baseline acute injuries excluded at enrolment	Prediction results (percent) for joint strain/sprain/muscle strain across six experimental groups; ‘acute sports injury rate’ compared between low-risk and high-risk groups; CNN loss trajectory	Model outputs from computer vision classification; injury-rate comparison described but method of injury ascertainment not detailed	Six experimental groups (Group 1–6); high-risk vs. low-risk groups; corrective training vs. no intervention (implied)

### 3.3 Risk of bias assessment

Table 3 summarizes the risk-of-bias appraisal of the 12 included studies using the QUIPS tool across the six standard domains (study participation, study attrition, prognostic factor measurement, outcome measurement, confounding, and statistical analysis/reporting), together with the overall judgement for each study to support interpretation of the strength of the available evidence.

**Table 3:** Risk of bias assessment.

Study	Participation	Attrition	Prognostic factor measurement	Outcome measurement	Confounding	Statistical analysis & reporting	Overall RoB
Abalo-Núñez et al. (36)	Moderate	Moderate	Moderate	High	High	High	High
Abalo-Núñez et al. (29)	Moderate	Moderate	High	High	High	Moderate	High
Abalo et al. (34)	Moderate	Moderate	Moderate	High	High	High	High
Gonçalves et al. (27)	Moderate	Moderate	High	High	High	Moderate	High
Hassmannová et al. (30)	Moderate	Moderate	High	High	High	Moderate	High
Hassmannová et al. (31)	Moderate	Moderate	High	High	High	Moderate	High
Purnell et al. (33)	Moderate	Low	High	High	High	Moderate	High
Abalo et al. (28)	Moderate	Moderate	High	High	High	Moderate	High
Li et al. (38)	High	High/Unclear	High	High	High	High	High
Sastre-Munar et al. (32)	Moderate	Low	Moderate	High	High	Moderate	High
Xiong et al. (40)	High	High/Unclear	High	High	High	High	High
Zhu et al. (39)	High	High/Unclear	High	High	High	High	High

### 3.4 Results of individual studies

Table 4 synthesizes studies examining athlete-related screening and prediction factors for injury in aerobic gymnastics–related populations. This table is organized around evidence on physical and biomechanical characteristics and risk-prediction approaches, including regression-based screening models and emerging machine-learning/early-warning systems. It includes studies that primarily evaluated individual-level attributes and/or algorithms intended to classify injury risk or discriminate injured versus uninjured gymnasts.

**Table 4:** Athlete-related screening and prediction factors (physical/biomechanical/functional and model-based risk prediction).

Study	Classification	Primary results	Main finding
Abalo-Núñez et al. (36)	Correlate of past/current injury	In gymnasts, injured vs. uninjured differed for right Q angle (*p* = 0.005), left Q angle (*p* = 0.003), and right-leg bilateral weight-bearing (*p* = 0.025); left-leg weight-bearing showed a non-significant trend (*p* = 0.056). A multivariable logistic model selecting left Q angle (LQA), right-leg weight-bearing (BWBRL) and left thigh perimeter (PLT) achieved 82.4% overall correct classification (specificity 89.7%, sensitivity 58.3%). An interaction model indicated the association of LQA with injury varied by body weight; reported prediction equation: P(injury) = 1/(1 + exp.(−(−24.95 + 1.624·LQA + 0.392·weight − 0.026·LQA × weight))), with best-reported AIC among compared models 45.47.	Excessive Q angle (especially the left Q angle) was associated with higher injury probability, and this relationship depended on gymnast body weight, supporting anthropometrics (alignment/loading distribution) as potential injury-predisposing factors.
Abalo et al. (34)	Predictor of future injury	Among gymnasts, 14 injuries occurred during the season; most frequent injury type was muscular (42.86%). Injured vs. uninjured gymnasts showed significant differences in mean (records 1–2) right and left Q angle and bilateral support weight (right/left) (reported as discriminatory in gymnasts but not controls). A logistic regression “predictor” model was reported with high specificity (90%) and low sensitivity (41.7%); equation presented as: P(Y = 1) = 1/(1 + exp.(5.323–0.272·MR1R2AQI)), where MR1R2AQI represents mean record 1–2 left Q angle.	Anthropometric/alignment variables (notably Q angle and weight-bearing measures) were associated with injury in aerobic gymnasts, but the proposed screening model showed limited sensitivity despite high specificity.
Li et al. (38)	Correlate of past/current injury	The proposed AI-based early warning framework (thermal monitoring + deep learning) was evaluated against comparator models. KO-DNN showed the best performance (accuracy 86.67%, MSE 90.6736, Pearson correlation 0.8994747) compared with RandomForest (accuracy 82.24%, Pearson 0.8824121), DNN (accuracy 81.34%, Pearson 0.8634734), Adaboost (accuracy 74.86%, Pearson 0.8487328) and Ridge regression (accuracy 69.43%, Pearson 0.789966). LightGBM+XGBoost fusion model with accuracy 81.74%, precision 73.49%, recall 61.69% (vs LinearRegression accuracy 68.54%).	AI-assisted infrared thermal monitoring paired with machine-learning classification demonstrated higher predictive performance (accuracy/error/correlation) than conventional comparator models for an aerobics injury early-warning task, supporting feasibility of real-time thermal anomaly–based risk flagging.
Xiong et al. (40)	Correlate of past/current injury	Algorithm-development study proposing a big-data + deep learning approach using FMS (7-test battery; total 0–21; score <14 described as higher injury risk) integrated with a neural network/CNN framework. Study reports selecting 200 college students as experimental subjects and grouping by aerobics level/sex. The model’s predicted injury-risk curve is reported as closely tracking the “true” curve, and an ablation comparison indicates that removing FMS meaningfully reduced predictive performance.	Incorporating FMS inputs into the deep learning architecture was reported to improve injury-risk prediction compared with a model variant without FMS (evidence presented primarily as visual fit/ablation plots rather than quantitative performance measures).
Zhu et al. (39)	Correlate of past/current injury	Algorithm-development study proposing a dual-branch injury-risk prediction model combining big-data feature analysis with computer vision (CNN) to recognize movement-related injury patterns (joint strain/sprain; muscle strain). In the vision experiment, training was conducted over 1 week with 50 sets of test actions. CNN loss decreasing to <0.1 after >900 steps (training curve). Reported model outputs for injury-type prediction across six groups ranged 69–80% for joint strain, 61–79% for joint sprain, and 69–79% for muscle strain.	A big-data and computer-vision framework was reported to achieve moderate–high percentage performance for classifying/predicting strain/sprain/muscle strain and to discriminate higher- vs. lower-risk group injury rates (supporting its use as an injury-risk prediction/early warning approach in aerobics gymnasts).

Table 5 summarizes studies reporting injury burden and training- or context-related correlates, including epidemiological descriptions of injury/pain prevalence, anatomical distribution and severity, as well as associations with training exposure, competition density, technical training context, and selected psychosocial or prevention/healthcare factors. This table captures the descriptive and explanatory evidence base most directly aligned with exposure and contextual determinants of injury risk and burden in aerobic gymnastics.

**Table 5:** Injury burden, training exposure, and contextual/psychosocial correlates (epidemiology and training-related factors).

Study	Classification	Primary results	Main finding
Abalo et al. (28)	Correlate of past/current injury	Injury prevalence: 38/42 (90.47%) reported at least one injury (senior category contributed the larger share). Injury severity: moderate 84.21%, mild 10.53%, severe 2.63%, very severe 2.63%. Injury types were most commonly muscular and articular, with frequent specific diagnoses including lower-limb sprain (18.42%), distension/strain (18.42%), and muscle fiber tear (15.79%). Anatomical distribution: lower limb 65.78%, upper limb 21.06%, trunk 13.15%. Timing/context: injuries occurred during multiple parts of sessions, with the highest index linked to difficulty elements; overall injury index was higher in training than competition. Preventive/management factors: 35.5% used protective materials; the proportion injured was higher among those not using protection. 44.13% reported medical consultation and required rehabilitation and had sequelae; 68% required physiotherapy/rehabilitation services.	In Spanish competitive aerobic gymnasts, injuries were very frequent, predominantly moderate, and mainly affected the lower limb with muscle/joint involvement; injury burden was linked to limited use of protective equipment and training on non-ideal surfaces, with most injuries occurring during training rather than competition.
Abalo-Núñez et al. (29)	Predictors of future injury	10 injuries were reported during the season (25% injured). Injury typology: ligamentous 40%, tendon 30%, muscular 30%, articular 0%. Injury location: upper limb 50% and lower limb 50%. All injuries occurred during specific technical training; ~50% occurred after jumps. Training associations: significant relationships were reported between number of injuries and experience, training days, and number of competitions (*p* < 0.05); and injured vs. uninjured differed by number of competitions (*p* < 0.05).	Higher training/competition exposure (experience, training days, competitions) was associated with greater injury occurrence, with many injuries arising during technical skill acquisition, particularly jump elements.
Gonçalves et al. (27)	Correlate of past/current injury	72.3% of athletes reported injury in the previous 12 months (mean 1.6 ± 1.5 injuries, typically 1–3 episodes). Injuries were most often muscle injuries (reported as 85.8% within muscle/tendon category) and joint sprains (69.2% within ligament/joint-capsule category); bone stress injuries were also frequent (47.5% within bone category). Mechanisms: acute 42.7%, overuse 34.4%, acute+overuse 22.9%; occurred mainly during training (74.6%), typically non-contact/alone (80.9%). Body regions: lower limbs most affected (notably ankle/knee/foot), followed by upper extremity and trunk. Reported risk-factor associations with injury status included psychological stress (*p* = 0.043) and concerns/usage related to individual protection equipment (reported as significant, *p* = 0.002).	In elite world-level aerobic gymnasts, injuries were highly prevalent and predominantly involved lower-limb muscle/joint tissues, occurring mainly during training; psychological stress and issues around individual protective equipment were associated with injury presence, supporting multifactorial prevention that includes stress-coping strategies and equipment/surface considerations.
Hassmannová et al. (30)	Correlate of past/current injury	98% of participants reported at least one musculoskeletal problem (painful condition and/or injury) during elementary school age. Most susceptible region was lower limbs, particularly knees and ankles; multiple concurrent problems were more common than a single problem. Prevalence: pain 86%, injury 48%, pain+injury 34% (multiple responses possible). Performance-level relationship: non-medal gymnasts had a higher injury occurrence than medal gymnasts; difference reported as statistically significant (*p* = 0.049). Care/prevention: immediate professional care after problem onset was sought by <29%; primary physiotherapy prevention 4%; secondary prevention <18%.	Musculoskeletal pain and injuries were nearly universal in elite school-aged gymnasts and concentrated in the lower limbs; poorer competitive performance (non-medal placing) was associated with higher injury occurrence, alongside very limited uptake of physiotherapy prevention/care.
Hassmannová et al. (31)	Correlate of past/current injury	Musculoskeletal problems occurred in 98% of gymnasts; lower limbs were most affected (reported frequency up to 140% reflecting multiple problems per athlete). Within lower-limb problems, ankles 40% and knees 36% were most frequent; back problems 25% and upper-limb problems 7% were reported. Pain was more frequent than injury: pain 86%, injury 48%, pain+injury 34%. Injuries were reported more often by gymnasts attaining non-medal positions than medal-winning gymnasts; the higher injury incidence in non-medal gymnasts was statistically significant (p = 0.049). Professional monitoring was limited: <29% sought immediate medical/physiotherapy care; primary physiotherapy prevention 4%; secondary prevention <18%.	Elite elementary-school-aged gymnasts most commonly experienced lower-limb (ankle/knee) pain and injuries, with worse competitive outcomes associated with higher injury burden; limited access/use of structured physiotherapy care may contribute to persistent or multiple problems.
Purnell et al. (33)	Correlate of past/current injury	Injury occurrence: 57.5% reported an acrobatics-related injury history; 50.7% reported an injury in the past 12 months; 28.8% had a current chronic injury. In the prior year, 86 injuries occurred over 29,209 training hours, giving 2.94 injuries/1000 h and 1.21 injuries per acrobat per year. Injury sites were predominantly knee, ankle and wrist, and most recent injuries occurred mainly during training (75.7%) with 58.8% during group skills. Training-associated thresholds (ROC): at age 11, training >8 h/week discriminated injured vs. uninjured with AUC 0.91, sensitivity 0.909, specificity 0.111, *p* = 0.002; thresholds at ages 12–15 were 8.5, 8.5, 9.75, 12.75 h/week, respectively, with AUCs 0.79–0.75 (*p*-values 0.037–0.067).	In acrobatic gymnastics, injury occurrence was common and concentrated in knee/ankle/wrist; the adolescent period was a critical window, with higher injury risk associated with higher weekly training loads (notably >8 h/week at age 11) and older age groups (≥13 years).
Sastre-Munar et al. (32)	Correlate of past/current injury	Retrospective end-of-season survey (*n* = 160 gymnasts). Injury prevalence: 80/160 (50.0%) reported injury during the 2020–2021 season, totaling 106 injuries; most frequent injury sites were ankle (25.5% of injuries; 16.9% of gymnasts), knee (14.2%; 9.4%), and low back (10.4%; 6.9%). Pain prevalence: 119/160 (74.4%) reported current pain; most frequent painful locations were low back (35.8%), knee (19.1%), ankle (16.2%), and wrist (9.8%). Between disciplines: rhythmic gymnasts had higher low-back pain prevalence (*p* = 0.003) and artistic gymnasts higher wrist pain prevalence (*p* = 0.011). Training/experience associations: injured gymnasts trained more hours/week than uninjured (19.9 ± 13.4 vs. 15.4 ± 11.5; *p* = 0.026; d = 0.36). Gymnasts with current pain were older (17.3 ± 3.0 vs. 15.5 ± 2.3; *p* = 0.001) and had more experience (10.4 ± 4.1 vs. 8.0 ± 3.9 years; *p* = 0.001). Catastrophizing: mean PCS total 21.4 ± 10.1; injured gymnasts had higher PCS (24.9 ± 9.4 vs. 17.8 ± 9.4; *p* < 0.001). Peak pain intensity correlated with number of painful locations (r = 0.75; *p* < 0.001) and with PCS (r = 0.42; *p* < 0.001).	Injuries and current pain were highly prevalent; higher training volume (hours/week) was associated with injury, and higher age/experience with current pain. Pain catastrophizing was higher in injured gymnasts and positively associated with pain burden.

## 4 Discussion

Across the included studies, the evidence indicates a high burden of musculoskeletal problems in aerobic gymnastics–related contexts, with injuries and pain most frequently affecting the lower limbs (particularly the ankle and knee) and, in some samples, the lumbar region. Across studies that examined correlates of injury, higher exposure (training volume and/or competition density) was associated with injury occurrence in multiple studies, although findings were not uniform across all designs and definitions. Lower-limb alignment/loading-distribution characteristics (e.g., Q-angle and bilateral weight-bearing measures) differed between injured and uninjured gymnasts in specific cohorts, but proposed screening models showed limited sensitivity. Additional domains emerged, including psychosocial correlates and limited prevention/healthcare uptake, alongside early-stage prediction approaches whose field-readiness remains constrained by study design and reporting limitations.

### 4.1 Injury burden, training exposure, and contextual/psychosocial correlates

Injury burden and clinical presentation were consistently high where adult competitive aerobic gymnasts were sampled, and lower, but still meaningful, in youth aerobic gymnasts. In world-level aerobic gymnasts, 72.3% reported at least one injury in the prior 12 months (mean 1.6 ± 1.5 injuries), with injuries occurring predominantly during training and typically via non-contact mechanisms (27). In Spanish championship aerobic gymnasts (junior and senior), 90.47% reported at least one injury, most often of moderate severity, with a predominance of lower-limb involvement (65.78%) and common diagnoses including sprain and muscle strain/tear patterns (28). In contrast, among younger aerobic gymnasts (9–17 years), 25% sustained injuries during a season (10 injuries in 40 athletes), suggesting that injury occurrence may scale with exposure and/or competitive demands across development and performance levels (29). Studies in broader gymnastics samples also emphasized high prevalence of musculoskeletal problems, particularly lower-limb pain/injury, reinforcing the likelihood that repetitive landing and impact exposures remain a dominant burden pathway across gymnastic sport participation (30–32).

The anatomical distribution and injury types reported across studies were coherent with the mechanical demands of aerobic gymnastics routines and training. Lower-limb involvement was prominent across elite aerobic gymnastics samples (27, 28) and in mixed gymnastics cohorts, where ankle and knee problems were particularly frequent (30, 31). In a multi-discipline sample, ankle injuries comprised 25.5% of injuries, with knee injuries and low-back injuries also prominent; pain prevalence was high (74.4%), with low back pain reported by 35.8% of gymnasts (32). These distributions are clinically relevant because ankle and knee injuries in jumping/landing sports often reflect modifiable loading patterns and neuromuscular control demands, while lumbar complaints may reflect cumulative spinal loading, technique, and fatigue management demands during repetitive extension/rotation tasks. Importantly, several studies also noted that injuries were more frequent in training than competition (27, 28), focusing attention on daily training content, repetition volume, and fatigue accumulation as core prevention targets.

Findings regarding when and how injuries occur further support a prevention focus on high-impact technical elements and the training environment. In youth aerobic gymnasts, all injuries were reported to occur during specific technical training, with approximately half occurring after jump elements (29). In Spanish championship aerobic gymnasts, injury indices were reported as higher during training than competition, and injuries were linked particularly to difficulty elements (28). In related acrobatic gymnastics data, most recent injuries occurred during training (75.7%), with many occurring during group skills (58.8%) (33). Although acrobatic gymnastics is not aerobic gymnastics, the convergence of “training-dominant” injury occurrence and skill-execution contexts across gymnastic sports suggests that prevention strategies should prioritize risk-managed progression of technical skill load, structured landing/jump mechanics coaching, and targeted load management during high-repetition technical blocks—particularly when athletes are exposed to fatigue and when training environments introduce additional constraints (e.g., surface characteristics, equipment).

Training exposure and workload-related factors were among the clearest domains linked to injury outcomes, consistent with the review’s second objective. In youth aerobic gymnasts, injury counts were significantly associated with experience, training days, and number of competitions, and injured versus uninjured athletes differed by competition number (34). In a broader gymnastics sample, injured gymnasts trained more hours per week than uninjured gymnasts (19.9 ± 13.4 vs. 15.4 ± 11.5; *p* = 0.026; d = 0.36), suggesting that absolute exposure may differentiate injury risk even when the directionality and confounding structure cannot be fully resolved (32). Critically, exposure (dose) and physical capacity/readiness likely interact, such that higher training volumes may increase injury risk primarily when the imposed load exceeds the athlete’s current fitness, tissue tolerance, and recovery resources (4). Contemporary workload–injury frameworks explicitly model this dual pathway, where workloads can increase risk via fatigue while simultaneously building protective fitness adaptations, implying that the same external dose may be harmful or protective depending on the athlete’s capacity and the rate of progression (4). Therefore, an apparent training volume and following injury association in retrospective or cross-sectional designs may be partially mediated or moderated by fitness (e.g., stronger/more fatigue-resistant athletes tolerate higher chronic loads with lower relative risk), while less-prepared athletes accrue disproportionate fatigue and tissue stress at the same volume (35). This supports prevention models that pair exposure monitoring with longitudinal capacity profiling (strength-endurance, power, landing control, and fatigue resistance), rather than attempting to define a single safe volume threshold across athletes.

Physical fitness and anthropometric/alignment correlates were most clearly represented by findings relating lower-limb alignment and load distribution to injury status in aerobic gymnasts. In two related aerobic-gymnastics cohorts, injured versus uninjured gymnasts differed on right and left Q-angle measures and on bilateral weight-bearing measures, suggesting that frontal-plane alignment and asymmetrical loading may be associated with injury occurrence (34, 36). For instance, Abalo-Núñez et al. (36) reported that Q-angle (particularly the left Q-angle) and right-leg bilateral weight-bearing contributed to injury classification performance, and that the association between left Q-angle and injury varied as a function of body weight—an interaction consistent with the concept that alignment-related tissue loading is magnified under higher absolute loads. However, despite these signals, the proposed models demonstrated limited sensitivity (e.g., 58.3 and 41.7%), implying substantial false-negative rates—i.e., many athletes who later sustain injury would not be flagged—so these tools should not be used for clearance, return-to-sport permissioning, or exclusion decisions (34, 36). This limitation is consistent with broader sports-injury screening critiques showing that even statistically significant risk factors often yield poor individual-level prediction because risk distributions overlap heavily between those who will and will not be injured (37).

Psychological and pain-related correlates emerged as an important complementary domain, indicating that injury risk and burden in aerobic gymnastics may be best interpreted using a biopsychosocial framework rather than a purely biomechanical one. In world championship aerobic gymnasts, psychological stress was associated with injury presence (*p* = 0.043) (27), and in a broader gymnastics cohort, injured gymnasts reported higher pain catastrophizing scores than uninjured gymnasts (PCS 24.9 ± 9.4 vs. 17.8 ± 9.4; *p* < 0.001), while catastrophizing correlated with peak pain intensity and pain distribution (32). Although the latter sample was not isolated to aerobic gymnastics, the convergence of stress-related and pain-coping constructs with injury/pain outcomes underscores a plausible pathway whereby psychological load interacts with physical load, influencing fatigue, recovery, symptom amplification, and reporting behavior.

The prevention and health-service utilization findings also have direct practical implications. In adolescent elite gymnasts sampled across gymnastic sports, immediate professional care after problem onset was sought by fewer than 29%, primary physiotherapy prevention was reported by only 4%, and secondary prevention uptake was under 18% (30, 31). In aerobic championship gymnasts, the proportion injured was higher among those not using protective materials, and a large proportion reported requiring physiotherapy/rehabilitation services (28). Similarly, in world-level aerobic gymnasts, variables related to individual protective equipment were associated with injury status (27). Data suggest that prevention capacity may be limited not only by training exposures but also by healthcare access pathways, education, and practical prevention resources (e.g., protective measures and surface considerations).

### 4.2 Physical/biomechanical/functional and model-based risk prediction

A further domain, predictive analytics and injury “early warning,” is developing rapidly but, within the included evidence base, remains insufficiently mature for confident applied use. Traditional regression-based models in aerobic gymnasts achieved high specificity but consistently low sensitivity, suggesting limited utility for identifying the majority of athletes who will go on to be injured (34, 36). Machine-learning studies reported comparatively higher classification performance metrics, including an infrared thermal imaging approach with accuracy values reported in the 80–87% range depending on the model (38), and computer-vision/big-data approaches reporting moderate-to-high percentage performance in classifying strain/sprain categories (39). Another approach emphasized Functional Movement Screen (FMS) integration and reported improved performance when FMS inputs were included, though results were presented primarily via qualitative curve comparisons rather than comprehensive validation measures (40). Despite their promise, these algorithmic studies typically lacked the transparency required for prognostic interpretation (e.g., clear injury definitions and ascertainment, dataset provenance, robust external validation, and confounder handling). Consequently, their findings should be viewed as hypothesis-generating and methodological demonstrations rather than field-ready injury-risk tools for aerobic gymnastics at present.

Across the included evidence base, the dominant methodological limitation was the frequent use of retrospective, self-reported ascertainment for both injury outcomes and candidate risk factors, which increases vulnerability to recall bias, exposure misclassification, and outcome misclassification. This was particularly evident in studies that collected injuries at the end of a season or across long recall windows using questionnaires/interviews (27–33, 36). Relatedly, several studies used convenience, event-based, or volunteer sampling, which may have introduced selection bias and limited external validity (28–32, 36). Moreover, there was meaningful heterogeneity in injury definitions and outcome operationalisation, ranging from time-loss/disruption thresholds to current or persisting problems and reconstructed historical pain/injury experiences, which likely reduced comparability and contributed to variability in observed associations (30–33, 36). A further limitation is that our database coverage did not include SPORTDiscus or CINAHL and because gymnastics and sports-medicine content can be indexed in those sources, some relevant discipline-specific studies may not have been captured despite reference-list screening.

A second cross-cutting limitation was the insufficient handling of confounding and limitations in analytic robustness, which restricts causal/prognostic inference and complicates translation into prevention practice. Multiple studies were largely descriptive or relied on bivariate association testing without clearly prespecifying and adjusting for key confounders such as prior injury, exposure time/training load, competitive level, and age/maturation (27–32). Where regression-based prediction was attempted, concerns remain regarding small sample sizes, retrospective outcome ascertainment, and model-building practices that increase the risk of overfitting and unstable estimates (33, 34, 36). In addition, several included studies were primarily algorithm-development studies in which the injury ground truth, dataset provenance, and validation strategy were insufficiently transparent for prognostic interpretation within an epidemiologic framework, limiting confidence in clinical or field deployment (38–40). These limitations stress the need for prospective injury surveillance with standardized outcome definitions, objective exposure quantification, prespecified confounder sets, and appropriately validated models to strengthen the certainty and usability of findings in aerobic gymnastics contexts. Future research should prioritize adequately powered prospective cohort surveillance in aerobic gymnastics using standardized injury definitions and exposure denominators, repeated in-season measurement of candidate risk factors, and transparent multivariable modeling with internal and external validation; promising algorithmic approaches (thermal imaging, computer vision, and deep learning) require rigorous dataset provenance, clear injury ascertainment, and real-world validation before applied adoption.

Minimum criteria for interpreting or translating prediction/early-warning models in aerobic gymnastics should include an explicit injury/label definition with ascertainment method and time window, prospective or near-real-time outcome capture where feasible, clear separation of training and test data with internal validation and reporting of calibration, and external validation in an independent sample prior to field deployment. For machine-learning models specifically, reporting should follow contemporary extensions for prediction models to ensure transparency about data provenance, model specification, and performance evaluation.

Future research should shift from single-factor association testing toward multifactorial, time-updated risk modeling that explicitly represents how exposure, fatigue, and fitness/readiness evolve across the season. This roadmap implies standardized prospective injury surveillance with clear case definitions and short recall windows, repeated in-season capture of training dose (volume, density, and change metrics) alongside capacity markers (strength-endurance, power, landing control) and recovery/context (sleep, stress, access to care), and multivariable models that report calibration and undergo internal/external validation before applied use.

Practical implications should be framed cautiously given the predominance of retrospective designs and heterogeneous injury definitions across the evidence base. Nevertheless, the available findings support prioritizing multifactorial prevention strategies that combine structured exposure monitoring (training hours, competition density, and abrupt load changes) with technique- and capacity-focused interventions targeting high-impact elements (jump/landing mechanics and fatigue-aware session design), because injuries were reported predominantly in training and commonly linked to technical/difficulty elements and higher exposure indices (27–29, 32, 33). Screening for lower-limb alignment/asymmetry (e.g., Q-angle and weight-bearing distribution) may contribute to individualized risk profiling, but should not be used in isolation given limited sensitivity of proposed models (34, 36). The consistent signals for psychosocial correlates (stress and catastrophizing) and low prevention/early-care uptake in youth samples also justify integrating psychologically informed education, early reporting pathways, and routine access to physiotherapy/medical oversight (27, 30–32).

## 5 Conclusion

In conclusion, this systematic review indicates that musculoskeletal injury and pain burden in aerobic gymnastics–related settings are substantial and are most frequently localized to the lower limbs, with injuries commonly occurring during training and in association with technical elements. Synthesizing across designs, the most defensible take-home message is that injury burden reflects the interaction between external exposure (the training/competition dose) and internal readiness (fitness, movement capacity, and recovery resources), rather than any single standalone risk. Across studies, higher exposure indices (training volume/competition density) and lower-limb alignment/loading-distribution measures (Q-angle and weight-bearing characteristics) were recurrently associated with injury status, while psychosocial factors and limited prevention/healthcare engagement may contribute to overall burden. In practical terms, prevention should therefore be multifaceted, namely including monitor and progress load, build capacity for high-impact elements, and strengthen recovery/reporting/clinical support pathways, consistent with dynamic injury-etiology models in which risk is recursive and changes over time. However, confidence in causal inference and generalizability is constrained by reliance on retrospective self-report and methodological heterogeneity, and current predictive models (statistical or machine-learning) should be considered preliminary until prospectively validated in representative aerobic-gymnast samples. Thus, injury prevention in aerobic gymnastics should be implemented as a multifactorial system that jointly manages training dose (volume, density, abrupt changes), develops physical readiness (strength, neuromuscular control, landing capacity), and strengthens recovery/context supports (stress, sleep, care pathways, surfaces/equipment), rather than relying on single screening thresholds.

## Data Availability

The original contributions presented in the study are included in the article/supplementary material, further inquiries can be directed to the corresponding author.
